# Diagnostic Performance of CBCT in Detecting Different Types of Root Fractures with Various Intracanal Post Systems

**DOI:** 10.3390/tomography11100116

**Published:** 2025-10-21

**Authors:** Serhat Efeoglu, Ecem Ozgur, Aysenur Oncu, Ahmet Tohumcu, Rana Nalcaci, Berkan Celikten

**Affiliations:** 1 Department of Oral Maxillofacial Radiology, Faculty of Dentistry, Ankara University, Ankara 06100, Türkiye; 2 Department of Endodontics, Faculty of Dentistry, Ankara University, Ankara 06100, Türkiye; 3 Department of Oral Maxillofacial Radiology, Faculty of Dentistry, Adıyaman University, Adıyaman 02040, Türkiye

**Keywords:** cone-beam computed tomography, root fracture detection, intracanal post systems, diagnostic accuracy

## Abstract

Objective: This study aimed to evaluate the diagnostic accuracy of two cone beam computed tomography (CBCT) devices using 18 imaging modalities in detecting root fractures—vertical, horizontal, and oblique—in teeth with intracanal post systems. Materials and methods: Ninety-six were extracted; single-rooted mandibular premolars were endodontically treated and restored with Bundle, Reforpost, or Splendor Single Adjustable posts. Controlled fractures of different types were induced using a universal testing machine. Each tooth was scanned with NewTom 7G and NewTom Go (Quantitative Radiology, Verona, Italy) under nine imaging protocols per device; varying in dose and voxel size, yielding 1728 CBCT images. Three observers (a professor of endodontics; a specialist; and a postgraduate student in endodontics) independently evaluated the images. Results: Observers demonstrated almost perfect agreement (κ ≥ 0.81) with the gold standard in fracture detection using NewTom 7G. No significant differences were found in sensitivity, specificity, or accuracy across voxel size and dose parameters for both devices in detecting fracture presence (*p* > 0.05). Similarly, both devices displayed comparable performance in identifying horizontal and oblique fractures (*p* > 0.05). However, in NewTom Go, regular and low doses with different voxel sizes showed reduced sensitivity and accuracy in detecting vertical fractures across all post systems (*p* ≤ 0.05). Conclusions: NewTom 7G, with its advanced detector system and smaller voxel sizes, provides superior diagnostic accuracy for root fractures. In contrast, NewTom Go displays reduced sensitivity for vertical fractures at lower settings. Clinical relevance: CBCT device selection and imaging protocols significantly affect the diagnosis of vertical root fractures.

## 1. Introduction

Cone-beam computed tomography (CBCT) has become a critical imaging modality in dentistry, providing high-resolution, three-dimensional (3D) images with relatively low radiation doses. However, the radiation dose associated with CBCT systems remains considerably higher than that of conventional two-dimensional (2D) imaging methods such as periapical or panoramic radiographs [1]. Anatomical superimpositions and reduced diagnostic sensitivity are often limitations of traditional two-dimensional radiographic techniques such as periapical and panoramic imaging. The CBCT offers the advantage of 3D examinations in overcoming these limitations [2].

Previous studies have evaluated the diagnostic performance of CBCT devices in detecting root fractures using different systems and imaging protocols.

These studies have demonstrated that CBCT provides higher diagnostic accuracy compared to conventional two-dimensional radiographic techniques. However, to the best of our knowledge, this is the first study to compare the imaging efficiency and diagnostic accuracy of the NewTom 7G and NewTom Go systems under standardized conditions. Root fractures, classified as vertical, horizontal, or oblique, pose a significant diagnostic challenge, particularly in endodontically treated teeth [3,4]. These fractures may occur because of trauma and masticatory loads, as well as excessive mechanical stress during root canal instrumentation, obturation, or post-space preparation [2]. Therefore, radiographic detection of root fractures is difficult due to the lack of definitive clinical or radiographic findings; fractures may mimic symptoms of periodontal or endodontic failure [2,5].

Although CBCT is known to show diagnostic sensitivity, artifacts caused by high-density materials such as root canal fillings and restorative materials, especially beam hardening, may negatively affect its sensitivity [6]. Previous studies indicated that these artifacts may obscure fracture lines or produce false-positive interpretations [7,8]. The severity of artifacts may vary depending on the composition of the intracanal post material [2,9].

Fiberglass posts have become a popular alternative to metal and cast posts. Their physical properties are similar to natural tooth dentin [10]. Splendor single adjustable post (SAP) (Angelus Odontologia, Londrina, PR, Brazil) has been developed as a novel fiber post design that incorporates a universal sleeve for better canal adaptation [11]. The Bundle Post System (Biolight Plus, Bio Composants Médicaux, Saint-Maur-des-Fossés, France), another type of fiber post system consisting of a bundle of thin individual posts, was introduced to adapt to atypical canal anatomies [12].

Although CBCT is widely used in endodontic diagnosis, there is a need, with the development of intracanal post systems, to evaluate the combined effects of device type, voxel size, exposure parameters, and post material on the detection accuracy of different types of root fractures. Therefore, the aim of this study is to evaluate the diagnostic accuracy of two CBCT devices using 18 distinct imaging protocols in detecting vertical, horizontal, and oblique root fractures in mandibular premolars restored with three contemporary intracanal post systems.

## 2. Materials and Methods

### 2.1. Study Design and Sample Preparation

This study was performed with the approval of the Ankara University Faculty of Dentistry Ethics Committee for Non-clinical Scientific Studies (Approval No.: 9/4; Date: 5 May 2025). This diagnostic accuracy study was conducted and reported following the STARD 2015 checklist. Furthermore, the present study was performed according to the PRILE-2021 guidelines for reporting laboratory studies in endodontology. The power analysis was conducted using G*Power software version 3.1. The expected Kappa value was set at 0.60, with an alpha level of 0.05 and a targeted power of 0.80. It was determined that the current sample size provides over 99% power under these parameters. This data indicates that the sample size is sufficient to reliably assess the observers’ ability to discriminate among the three fracture types meaningfully. Therefore, this study was conducted using 96 extracted lower premolar teeth. The teeth were obtained from a certified human tooth bank under ethical approval, and no extractions were performed specifically for this research. All samples were thoroughly cleaned and stored in distilled water at room temperature until use. Single-rooted premolars were chosen to ensure standardization and to minimize anatomical variability that could influence fracture detection accuracy. Multirooted teeth were excluded due to their complex canal morphology, which could act as a confounding factor.

The samples were initially divided into three main groups (*n* = 32) and restored with Bundle, Reforpost, and Splendor posts. Each group was then divided into four subgroups (*n* = 8) according to simulated fracture types: vertical, horizontal, oblique, and a control group with no fracture. All samples were subsequently scanned using nine different imaging protocols for each of the two CBCT devices. The power analysis for the agreement assessment was performed based on three observers, fracture presence/absence, and fracture type detection, using 1728 CBCT images. This evaluation plan allowed for the analysis of both inter-observer and intra-observer reliability.

Teeth were confirmed to be free of cracks or fractures, and an examination of canal anatomy was conducted by using micro-CT. The specimens that had round-shaped single -canal morphology were selected. After confirmation, all specimens were standardized by removing the crowns such that the root length was 13 mm. All root canals were prepared with the Revo S system (MicroMega, Besancon, France) up to the SU file and irrigated with 2.5% sodium hypochlorite (NaOCl) between each instrumentation. The final irrigation was applied with 5% EDTA, 2.5% NaOCl, and distilled water. The canals were dried with paper points. Then, the canals were filled using the single cone technique with a resin-based sealer (Adseal, Meta Biomed Co., Cheongju, Republic of Korea) and size 25/0.06 of gutta-percha cones (Pearl Dent., Bucheon, Republic of Korea). Two weeks later, the post space of samples was prepared, preserving 4 mm of gutta-percha at the root tip. The post space was rinsed with 2.5% NaOCl, 5% EDTA, 2.5% NaOCl again, and distilled water, then dried with paper points. The dual-cure adhesive resin cement (i-FIX Duo, i-Dental, Lithuania) was applied. Then, the Bundle, Reforpost (Angelus Odontologia, Londrina, Brazil), and Splendor posts were placed into the root canal according to the manufacturer’s instructions, and each post was cured for 20 s with an LED light device (Woodpecker DTE O-Light Plus, Guilin, China). After root canal treatment and post placement procedures, micro-cracks or fractures were examined using micro-CT. No significant cracks or fractures were observed in any of the samples, and the very minimal micro-cracks detected were considered negligible. Eight teeth per group were left as control samples, and the other teeth were fractured along vertical, horizontal, and oblique lines.

### 2.2. Fracture Induction

**Figure 1:**
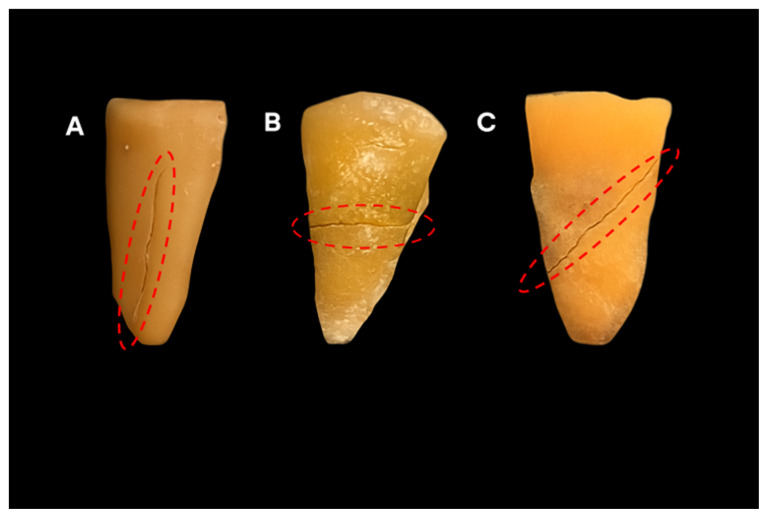
Representative photographs of the roots following fracture induction. The red circles indicate the regions of interest where the structural alterations were evaluated. (**A**): Vertical fracture line, (**B**): Horizontal fracture line, (**C**): Oblique fracture line.

A superficial groove was opened with a diamond disc in the middle 1/3 of the root, to create a horizontal and oblique fracture. For a vertical fracture, a groove is created on the coronal surface of the root. Root fractures were artificially induced using a universal testing machine (Lloyd Instruments, Fareham, England) under controlled conditions. A tapered metal tip was placed in the notch and guided into the canal at a speed of 1 mm per minute, and it was programmed to stop automatically when a fracture occurred. Fractures were produced mechanically by applying a special metallic tip. The testing machine was set to apply a maximum load of 2 kN. This force was sufficient to create a root dentin fracture without fragmentation [13]. The fracture types were verified under ×10 magnification using a dental operating microscope (Carl Zeiss, Oberkochen, Germany). The two halves of the same tooth were then bonded together with cyanoacrylate adhesive. Figure 1 displays vertical, horizontal, and oblique fracture lines of roots.

### 2.3. CBCT Imaging and 3D Radiographic Evaluation

**Figure 2:**
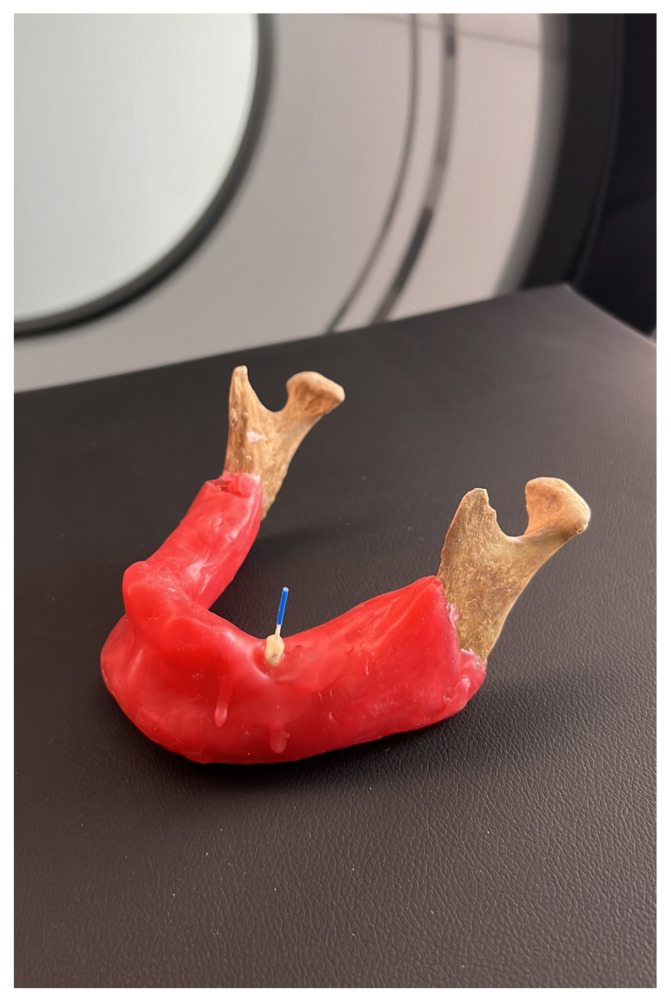
The image shows that the tooth sample is placed in the mandibular bone.

Subsequently, A single dry human mandible was used for all CBCT scans to maintain uniform imaging conditions. The mandible was mounted on a custom-made acrylic holder and positioned so that the occlusal plane was parallel to the floor and perpendicular to the X-ray beam, in accordance with the manufacturers’ clinical positioning recommendations (Figure 2). The alveolar sockets were artificially created by the researcher to simulate clinical conditions and to ensure standardized positioning of the teeth within the mandible. Each socket was prepared using a low-speed bur to a standardized size and depth, following the natural anatomical contours of the mandible. During this process, the cortical integrity of the bone was preserved, and only minimal shaping was performed to allow for stable placement of the teeth. In this study, soft modeling wax (Cavex Set Up Wax, Cavex, Haarlem, The Netherlands) was used to stabilize the teeth within the alveoli. This material was selected because it provides sufficient stabilization without creating radiopaque artifacts or affecting image quality, as supported by previous in vitro studies. Scanned with NewTom 7G and NewTom Go CBCT devices (Quantitative Radiology, Verona, Italy) at a standardized field of view (FOV) of 60 × 60 mm. Each device employed three dose settings (low, standard, and best quality), resulting in voxel sizes ranging from 80 µm to 300 µm. In the NewTom 7G device, the low-dose protocol was set at 100 kVp, 10.08 mA, and 1.4 s; the regular-dose protocol at 110 kVp, 20.16 mA, and 2.9 s; and the best-quality dose protocol at 120 kVp, 41.6 mA, and 4.2 s. In the NewTom Go device, the low-dose protocol was set at 90 kVp, 6.4 mA, and 1.6 s; the regular dose at 90 kVp, 9.6 mA, and 2.4 s; and the best-quality dose at 90 kVp, 22.4 mA, and 5.6 s.

**Figure 3:**
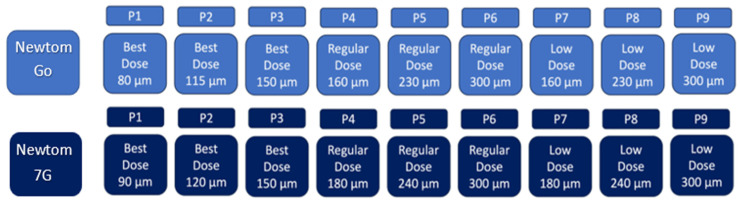
The imaging modalities included in this study were labeled P1 to P9.

**Figure 4:**
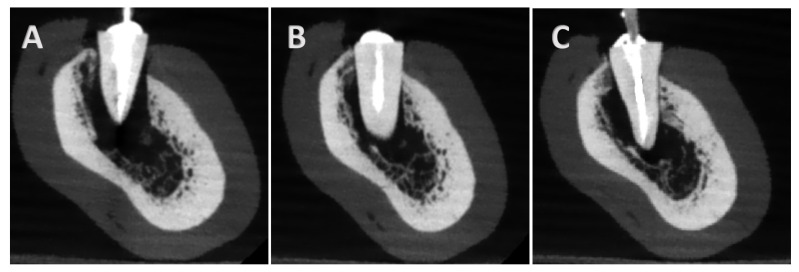
The images indicate no fracture samples. (**A**): Bundle control sample, (**B**): Reforpost control sample, (**C**): Splendor control sample.

**Figure 5:**
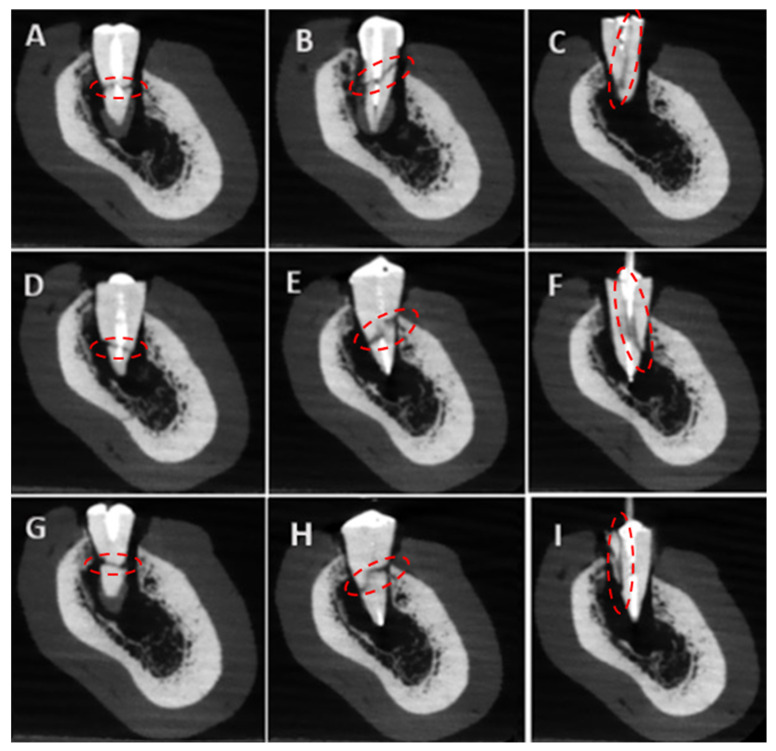
The representative images obtained with the NewTom 7G device: The red circles indicate the regions of interest where the structural alterations were evaluated. (**A**): Bundle-Horizontal, (**B**): Bundle-Oblique, (**C**): Bundle-Vertical, (**D**): Reforpost-Horizontal, (**E**): Reforpost-Oblique, (**F**): Reforpost-Vertical, (**G**): Splendor-Horizontal, (**H**): Splendor-Oblique, (**I**): Splendor-Vertical.

**Figure 6:**
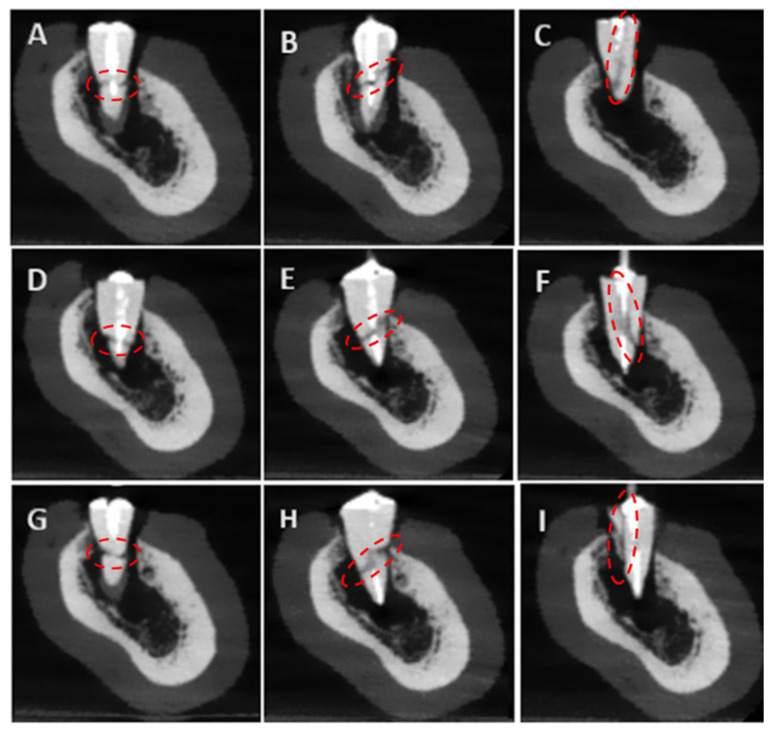
The representative images obtained with the NewTom GO device; The red circles indicate the regions of interest where the structural alterations were evaluated. (**A**): Bundle-Horizontal, (**B**): Bundle-Oblique, (**C**): Bundle-Vertical, (**D**): Reforpost-Horizontal, (**E**): Reforpost-Oblique, (**F**): Reforpost-Vertical, (**G**): Splendor-Horizontal, (**H**): Splendor-Oblique, (**I**): Splendor-Vertical.

A total of 18 imaging protocols (9 per device) were used, producing 1728 CBCT scans. Figure 3 presents the experimental group’s baseline parameters. All scans were saved in DICOM format and evaluated in a double-blind manner by three observers: a professor of endodontics with 10 years of experience, a specialist with 2 years of experience, and a postgraduate student in endodontics. Images were assessed twice at a 1-week interval in axial, coronal, and sagittal planes for fracture detection. All observers examined all images for the presence or absence of fractures. If present, they classified the fracture type as horizontal, vertical, or oblique. Figure 4 presents CBCT images of the control samples without fracture lines, with one representative image displayed for each post system. Figure 5 and Figure 6 present the images captured using the NewTom 7G and GO devices, respectively.

### 2.4. Statistical Analysis

All analyses were performed using the Jamovi software 2.7.8 (Sydney, Australia). Interobserver and intraobserver reliability were assessed using Cohen’s Kappa statistics because of the categorical outcomes involving the classification of fracture presence and fracture type. Agreement with the gold standard was calculated separately for each of the three observers. Kappa values were interpreted according to Landis and Koch’s criteria: ≤0.20 as slight, 0.21–0.40 as fair, 0.41–0.60 as moderate, 0.61–0.80 as substantial, and ≥0.81 as almost perfect agreement. The diagnostic sensitivity, specificity, and accuracy were evaluated for each image modality. The statistical differences between imaging modalities were assessed using the Cochran’s Q test. When significant differences were identified, pairwise McNemar tests were performed to determine between which groups the differences existed.

## 3. Results

Interobserver reliability Kappa values of agreement between observers and the gold standard in determining the presence or absence of fracture in teeth treated with Reforpost, Splendor SAP, and Bundle post systems are presented in Table 1. In all three post systems, the professor, specialist, and student displayed almost perfect agreement with the gold standard situation (κ ≥ 0.81). Observers with different experience levels were able to detect the presence or absence of root fracture in images obtained with both devices.

Table 2 presents the interobserver agreement Kappa values of agreement between observers and the gold standard, which determine the fracture type when different post-restoration methods were employed to detect horizontal, vertical, and oblique fractures. In all three post systems, all observers displayed almost perfect agreement with the gold standard situation in the Newtom 7G device. However, all observers displayed substantial agreement with the gold standard situation in the Newtom GO device. Intra-observer reliability showed high agreement in all three observers for all assessments (professor κ = 0.965, specialist κ = 0.940, postgraduate student κ = 0.942).

The mean diagnostic values for accuracy, sensitivity, and specificity of the Newtom 7G and GO devices, based on three observer evaluations using imaging modalities, are presented in Table 3 and Table 4 for the detection of fracture presence and Table 5 and Table 6 for the determination of fracture type. No statistically significant difference was observed in terms of diagnostic sensitivity, specificity, and accuracy of voxel size and dose parameters in detecting the presence or absence of fractures in both devices (*p* > 0.05). There was no statistically significant difference observed in terms of diagnostic sensitivity, specificity, and accuracy of both devices in determining the type of horizontal and oblique fracture (*p* > 0.05). However, Newtom GO devices, which employed a low and regular dose mode, demonstrated lower sensitivity and accuracy values in detecting vertical fractures across all three post types (*p* ≤ 0.05).

## 4. Discussion

Vertical, oblique, and horizontal fractures may occur in vital teeth or endodontically treated teeth due to traumatic dental injuries and masticatory force [14]. In this in vitro study, the diagnostic accuracy of vertical, horizontal, and oblique root fractures induced in teeth restored with three different intracanal post systems was assessed through 18 distinct CBCT imaging protocols generated by NewTom GO and 7G devices, each with various dose and voxel size settings.

No statistically significant difference was displayed between the three observers with different levels of clinical experience in terms of their ability to detect the presence and type of fractures. All observers showed almost perfect agreement and diagnostic consistency with the gold standard as a result of evaluating all images obtained with the Newtom 7G device. However, almost perfect agreement was observed in the detection of fractures created with all post types in the evaluation of images obtained from the NewTom Go device. Substantial agreement was observed between observers and the gold standard in determining the fracture type. The Newtom 7G device demonstrated high diagnostic sensitivity, specificity, and accuracy values in detecting the presence of fractures and determining fracture type in all imaging modes. High diagnostic accuracy was observed in all modes of the Newtom Go device in detecting the presence of fractures and determining the type of horizontal and oblique fractures. On the other hand, lower sensitivity and accuracy values were observed in the detection of vertical fracture types in the low and regular dose modes of the Newtom Go device. Based on these findings, low-resolution images may create diagnostic difficulties in the detection of vertical root fracture types, and the effect of the imaging protocol on diagnostic performance may be more decisive than observer experience.

Excessive loss of coronal tooth structure is frequently observed in endodontically treated teeth. Post placement is generally indicated when no coronal walls are remaining to retain a core buildup [15]. To accurately simulate this clinical scenario and achieve standardization during imaging procedures, the crowns were removed, and the roots were used in this study. The Splendor SAP, Bundle, and Reforpost used in this study are post systems that can adapt to the post space with different materials and design characteristics. The potential of intracanal posts to cause root fractures has been reported in various studies in the literature [16,17]. The presence of post materials in root canals increases artifacts, such as beam hardening and scattering in CBCT images, thereby complicating fracture detection. This complication has been reported as one of the primary causes of diagnostic difficulty [18,19]. In addition to the restorative properties of the post material, its diagnostic suitability must also be considered.

The posts used in this study also differed in design and composition. Reforpost consists of glass fiber, pigmented epoxy resin, and stainless-steel filaments [20]. Due to the presence of metallic components (stainless-steel filaments), this system is more prone to beam hardening and streak artifacts on CBCT images. Splendor SAP is composed of a homogeneous, fiber-reinforced composite matrix [21] and, since it contains no metallic elements, exhibits a much lower tendency to produce artifacts. Bundle fiber posts, on the other hand, are made of glass fiber bundles shaped within a urethane dimethacrylate–based resin matrix and reinforced with radiopaque agents to enhance radiographic visibility [12]. This structure increases image contrast while keeping artifact formation at a limited level.

Although different post materials may produce various levels of imaging artifacts depending on their composition and density, our controlled in vitro results demonstrated that these differences did not significantly affect the fracture detection rates. However, in the presence of posts with a high tendency to generate artifacts, fracture detection by CBCT may become more difficult. In contrast, fiber-based post systems (particularly Splendor SAP and Bundle Fiber Posts) generate fewer artifacts and therefore offer a diagnostic advantage [22,23]. These findings suggest that the selection of post material should be based not only on mechanical performance but also on radiological behavior, as minimizing artifact formation can improve the diagnostic accuracy of CBCT assessments.

The present findings reinforced the value of CBCT as a reliable diagnostic method in detecting the presence of root fracture. In diagnosing fracture type, high sensitivity, specificity, and accuracy were observed for all imaging modalities in detecting horizontal and oblique fractures, and no statistically significant difference was found for both devices. The sensitivity and accuracy values obtained in the low and regular modes of the NewTom Go device and in the protocols created with different voxels were low, preventing observers from diagnosing the vertical fracture type. In contrast, in images obtained with the NewTom 7G device, the presence of fractures and fracture types can be detected at a higher rate in all imaging protocols created using different voxel sizes in low, regular, and best modes. This ability is attributed to the device’s smaller voxel sizes, advanced imaging technology, and high-resolution reconstruction capacity [24]. This difference suggests that the device’s technical specifications are decisive in detecting lesions that are difficult to identify, such as fine anatomical structures and vertical fractures.

The image quality of CBCT devices is directly related to technical parameters such as the dose received by the patient, voxel size, and detector technology [25,26]. Additionally, the success of CBCT in detecting root fractures has been reported to be influenced by factors such as tube current (mA), field of view (FOV), and the presence of endodontic materials (e.g., gutta-percha, metal, or glass fiber posts) [27].

Among the factors affecting image quality, small voxel sizes generally increase spatial resolution and have been reported to facilitate the visualization of fracture lines; however, our findings indicate that reducing the voxel size below a certain threshold value (e.g., 80–90 µm) does not result in a significant improvement in diagnostic performance [28]. This result demonstrates that voxel size alone is not a definitive measure of diagnostic success. Diagnostic reliability may also be influenced by factors such as the anatomical location of the fracture, the type of intracanal post used, and the size of image artifacts [28]. Smaller voxel sizes may improve fracture detection, particularly in the presence of intracanal posts; however, some studies have reported that voxel size may have a limited effect under certain conditions [29,30].

It is known that different CBCT imaging protocols, such as high-resolution imaging and standard zoom settings, can affect the diagnostic value of fracture detection, particularly in endodontically treated teeth restored with fiber or screw-type posts [24]. Studies comparing different CBCT protocols have indicated that newer software versions and optimized protocols can provide acceptable image quality with lower radiation doses [31]. Although ultra-low-dose CBCT protocols may exhibit lower diagnostic accuracy for certain applications in fracture detection, they may still be beneficial in some clinical scenarios where low dose is critical [32].

To our knowledge, there are no studies in the literature that evaluate and compare the diagnostic accuracy of NewTom Go and NewTom 7G CBCT devices. The NewTom 7G and NewTom Go devices used in this study also show differences in terms of these parameters. In particular, the NewTom 7G device has enabled the acquisition of high-resolution images owing to its more advanced detector system and smaller voxel sizes [33]. According to the current results, the success of Newtom 7G in terms of diagnostic accuracy at all doses and voxel sizes can be attributed to these advantages.

Three different dose levels (low, regular, and best) and three different voxel sizes were used for each dose level on both devices. However, in the best mode protocols of the NewTom 7G, owing to the high spatial resolution offered by small voxel sizes, excellent agreement (Cohen’s kappa > 0.8) was achieved between the actual situation and the observers in determining both the presence of fractures and the type of fracture (oblique, horizontal, or vertical). This finding demonstrates that image quality directly affects not only the presence of fractures but also their morphological classification.

Vertical fractures are morphologically thin and often non-dislocated structures; so, in low-resolution protocols, the fracture line can be confused with post-source artifacts and cannot be reliably detected by observers [34]. This finding indicates that the limitations of low-dose protocols should be considered in the clinical decision-making process, especially in cases where vertical fractures are suspected.

### Regarding Limitations

This study was conducted under in vitro conditions, which may not fully replicate clinical environments, including factors such as patient movement, soft tissue scattering, and variations in bone density. Although only lower premolar teeth were used to ensure standardization, this limits the generalizability of the results to other tooth types. Furthermore, the use of a single dry human mandible may not represent the anatomical variability found in the general population. Although micro-CT imaging was used to verify the structural integrity of the samples, potential microstructural differences between in vivo and in vitro conditions cannot be completely eliminated. Future in vivo studies including a wider range of tooth types and anatomical variations are necessary to validate and extend these findings.

## 5. Conclusions

The present study revealed that CBCT devices and protocols showed significant differences in diagnostic accuracy in the detection of root fractures. Both the Newtom 7G and GO devices demonstrated high diagnostic performance in detecting the presence and determining the type of horizontal and oblique root fractures regardless of the post system used. However, the Newtom GO device showed significantly lower sensitivity and accuracy in detecting vertical fractures. Protocols using low doses and large voxel sizes may be insufficient, especially in distinguishing vertical fractures. Therefore, in cases where root fracture is suspected in clinically prosthetically restored teeth, selecting the appropriate CBCT protocol is crucial for accurate diagnosis and effective treatment planning. These findings are limited to premolar teeth and the specific in vitro conditions applied in this study. Therefore, further research using different tooth types and clinical conditions is recommended.

## Figures and Tables

**Table 1:** Interobserver reliability Kappa values between observers and the golden standard to detect the presence or absence of fracture, when different post-restoration methods were employed.

Post Systems	CBCT Devices	Professor	Specialist	Post-Graduate Student
Reforpost	Newtom 7G	0.932	0.877	0.902
Splendor SAP	Newtom 7G	0.887	0.862	0.860
Bundle Post	Newtom 7G	0.904	0.886	0.894
Reforpost	Newtom Go	0.935	0.880	0.905
Splendor SAP	Newtom Go	0.890	0.890	0.863
Bundle Post	Newtom Go	0.807	0.880	0.897

**Table 2:** Interobserver reliability Kappa values between observers and the golden standard to detect the horizontal, vertical, and oblique fractures, when different post-restoration methods were employed.

Post Systems	CBCT Devices	Professor	Specialist	Post-Graduate Student
Reforpost	Newtom 7G	0.875	0.874	0.872
Splendor SAP	Newtom 7G	0.871	0.868	0.870
Bundle Post	Newtom 7G	0.877	0.870	0.871
Reforpost	Newtom Go	0.715	0.616	0.720
Splendor SAP	Newtom Go	0.619	0.612	0.612
Bundle Post	Newtom Go	0.612	0.718	0.713

**Table 3:** Mean sensitivity, specificity, and accuracy values in the evaluation of fracture presence or absence detection with the Newtom 7G device according to the three observers’ assessments.

		Splendor SAP			Reforpost			Bundle		
Modality	Fracture Type	Sensitivity	Specificity	Accuracy	Sensitivity	Specificity	Accuracy	Sensitivity	Specificity	Accuracy
7G P1	presence	90.6	100	87.5	95.8	100	96.9	95.8	100	96.9
	absence	100	95.5	90.6	100	95.8	96.9	100	95.8	96.9
7G P2	presence	90.3	96.6	92.7	91.7	100	93.8	91.7	100	93.8
	absence	100	90.3	92.7	100	91.7	93.8	100	91.7	93.8
7G P3	presence	91.7	100	93.8	93	100	94.8	91.7	100	93.8
	absence	100	91.7	93.8	100	93	94.8	100	91.7	93.8
7G P4	presence	96	100	96.9	95.8	100	96.9	95.8	100	96.9
	absence	100	96	96.9	100	93	94.4	100	95.8	96.9
7G P5	presence	95.5	100	90.6	95.5	100	90.6	95.7	100	96.9
	absence	100	95.5	90.6	100	95.5	90.6	100	95.7	96.9
7G P6	presence	100	99	99.2	100	99	99.2	97.9	100	98.4
	absence	99	100	99.2	99	100	99.2	100	97.9	98.4
7G P7	presence	87.5	100	90.6	95.8	100	96.9	93	100	94.8
	absence	100	87.5	90.6	100	95.8	96.9	100	91.7	93.8
7G P8	presence	95.8	100	96.9	95.8	100	96.9	95.8	100	96.9
	absence	100	95.8	96.9	100	95.8	96.9	100	95.8	96.9
7G P9	presence	87.5	100	90.6	91.7	100	93.8	91.7	100	93.8
	absence	100	87.5	90.6	100	91.7	93.8	100	91.7	93.8

**Table 4:** Mean sensitivity, specificity, and accuracy values in the evaluation of fracture presence or absence detection with the Newtom GO device according to the three observers’ assessments.

		Splendor SAP			Reforpost			Bundle		
Modality	Fracture Type	Sensitivity	Specificity	Accuracy	Sensitivity	Specificity	Accuracy	Sensitivity	Specificity	Accuracy
GO P1	presence	83.3	100	87.5	88.9	100	91.6	91.7	100	93.7
	absence	100	83.3	87.5	100	88.9	91.6	100	91.7	93.7
GO P2	presence	84.7	100	87.5	100	87.5	90.6	91.7	100	93.7
	absence	100	81.8	87.5	87.5	100	90.6	100	91.7	93.7
GO P3	presence	89.3	100	91.7	87.5	100	90.6	92.9	100	94.4
	absence	100	89.3	91.7	100	87.5	90.6	100	92.9	94.4
GO P4	presence	96.4	100	97.2	85.7	100	88.9	89.3	100	91.7
	absence	100	96.4	97.2	100	85.7	88.9	100	89.3	91.7
GO P5	presence	92.9	100	94.4	96.4	100	97.2	92.9	100	94.4
	absence	100	92.9	94.4	100	96.4	97.2	100	92.9	94.4
GO P6	presence	93.1	100	94.4	96.4	100	97.2	95.2	100	96.2
	absence	100	93.1	94.4	100	96.4	97.2	100	95.2	96.2
GO P7	presence	92.9	100	94.4	88.1	100	89.8	100	96.4	97.2
	absence	100	92.9	94.4	100	88.1	89.8	96.4	100	97.2
GO P8	presence	89.3	100	91.7	92.9	100	94.4	92.9	100	94.4
	absence	100	89.3	91.7	100	92.9	94.4	100	92.9	94.4
GO P9	presence	89.3	100	91.7	92.9	100	94.4	92.9	100	94.4
	absence	100	89.3	91.7	100	92.9	94.4	100	92.9	94.4

**Table 5:** Mean sensitivity, specificity, and accuracy values evaluation on Newtom 7G device according to three observers’ assessments in detecting fracture type.

		Splendor SAP			Reforpost			Bundle		
Modality	Fracture Type	Sensitivity	Specificity	Accuracy	Sensitivity	Specificity	Accuracy	Sensitivity	Specificity	Accuracy
7G P1	Horizontal	87.5	100	96.9	87.5	95.8	93.8	87.5	100	96.9
	Vertical	87.5	95.8	93.8	87.5	100	96.9	87.5	95.8	93.8
	Oblique	87.5	91.7	90.6	87.5	91.7	90.6	100	91.7	93.8
7G P2	Horizontal	87.5	100	96.9	87.5	100	96.9	87.5	100	96.9
	Vertical	87.5	95.8	93.8	87.5	100	96.9	87.5	95.8	93.8
	Oblique	88.9	91.3	90.6	100	91.7	93.8	87.5	91.7	90.6
7G P3	Horizontal	87.5	95.8	93.8	87.5	100	96.9	87.5	95.8	93.8
	Vertical	87.5	95.8	93.8	87.5	95.8	93.8	87.5	91.7	90.6
	Oblique	87.5	95.8	93.8	87.5	91.7	90.6	87.5	100	96.9
7G P4	Horizontal	87.5	95.8	93.8	87.5	100	96.9	87.5	100	96.9
	Vertical	87.5	100	96.9	87.5	95.8	93.8	87.5	100	96.9
	Oblique	87.5	91.7	90.6	87.5	91.7	90.6	87.5	95.8	93.8
7G P5	Horizontal	87.5	100	96.9	87.5	100	96.9	87.5	100	96.9
	Vertical	87.5	95.8	93.8	87.5	95.8	93.8	87.5	91.7	90.6
	Oblique	87.5	91.7	90.6	87.5	91.7	90.6	87.5	95.8	93.8
7G P6	Horizontal	87.5	95.8	93.8	87.5	100	96.9	87.5	95.8	93.8
	Vertical	87.5	95.8	93.8	87.5	95.8	93.8	87.5	100	96.9
	Oblique	87.5	95.8	93.8	87.5	91.7	90.6	87.5	91.7	90.6
7G P7	Horizontal	87.5	100	96.9	87.5	100	96.9	87.5	100	96.9
	Vertical	87.5	91.7	90.6	87.5	95.8	93.8	87.5	95.8	93.8
	Oblique	87.5	100	96.9	87.5	95.8	93.8	87.5	95.8	93.8
7G P8	Horizontal	91.6	95.8	93.8	87.5	95.8	93.8	87.5	100	96.9
	Vertical	87.5	100	96.9	87.5	95.8	93.8	87.5	95.8	93.8
	Oblique	87.5	95.8	93.8	87.5	100	96.9	87.5	95.8	93.8
7G P9	Horizontal	87.5	100	96.9	87.5	100	96.9	87.5	100	96.9
	Vertical	87.5	95.8	93.8	87.5	95.8	93.8	87.5	95.8	93.8
	Oblique	87.5	95.8	93.8	87.5	95.8	93.8	87.5	100	96.9

*p* > 0.05 for all comparisons.

**Table 6:** Mean sensitivity, specificity, and accuracy values evaluation on Newtom GO device according to three observers’ assessments in detecting fracture type.

		Splendor SAP			Reforpost			Bundle		
Modality	Fracture Type	Sensitivity	Specificity	Accuracy	Sensitivity	Specificity	Accuracy	Sensitivity	Specificity	Accuracy
GO P1	Horizontal	87.5	95.8	93.8	87.5	100	96.9	87.5	95.8	93.8
	Vertical	87.5	95.8	93.8	100	95.8	96.9	87.5	100	96.9
	Oblique	87.5	95.8	93.8	87.5	95.8	93.8	100	95.8	96.9
GO P2	Horizontal	100	100	100	87.5	95.8	93.8	87.5	100	96.9
	Vertical	87.5	95.8	93.8	87.5	100	96.9	87.5	95.8	93.8
	Oblique	88.9	91.3	90.6	87.5	91.7	90.6	87.5	91.3	90.6
GO P3	Horizontal	100	100	100	87.5	100	96.9	87.5	95.8	93.8
	Vertical	87.5	95.8	93.8	87.5	95.8	93.8	87.5	95.8	93.8
	Oblique	87.5	95.8	93.8	87.5	91.7	90.6	87.5	95.8	93.8
GO P4	Horizontal	87.5	100	96.9	87.5	95.8	93.8	87.5	100	96.9
	Vertical	37.5 *	95.8	79.2 *	50 *	95.8	79.2 *	50 *	93.8	79.2 *
	Oblique	87.5	100	96.9	87.5	100	96.9	87.5	95.8	93.8
GO P5	Horizontal	87.5	100	96.9	87.5	100	96.9	87.5	100	96.9
	Vertical	37.5 *	95.8	79.2 *	50 *	95.8	79.2 *	50 *	93.8	79.2 *
	Oblique	88.9	91.3	90.6	87.5	91.7	90.6	87.5	95.8	93.8
GO P6	Horizontal	87.5	100	96.9	87.5	100	96.9	87.5	95.8	93.8
	Vertical	25 *	93.8	79.8 *	37.5 *	95.8	79.2 *	50 *	93.8	79.2 *
	Oblique	87.5	95.8	93.8	87.5	91.7	90.6	87.5	91.7	90.6
GO P7	Horizontal	87.5	100	96.9	87.5	100	96.9	87.5	100	96.9
	Vertical	25 *	93.8	79.8 *	37.5 *	95.8	79.2 *	50 *	93.8	79.2 *
	Oblique	87.5	100	96.9	87.5	95.8	93.8	87.5	95.8	93.8
GO P8	Horizontal	87.5	95.8	93.8	87.5	95.8	93.8	87.5	95.8	93.8
	Vertical	25 *	93.8	79.8 *	37.5 *	95.8	79.2 *	50 *	93.8	79.2 *
	Oblique	87.5	95.8	93.8	87.5	100	96.9	87.5	95.8	93.8
GO P9	Horizontal	87.5	95.8	93.8	87.5	100	96.9	87.5	100	96.9
	Vertical	25 *	93.8	79.8 *	37.5 *	95.8	79.2 *	50 *	93.8	79.2 *
	Oblique	87.5	91.7	90.6	87.5	95.8	93.8	87.5	100	96.9

The superscript asterisk indicates a statistical difference at the *p* < 0.05 level.

## Data Availability

The data can be provided by the corresponding author upon request of the readers due to (Due to the large file size of raw CBCT data in DICOM format).
